# Effectiveness and drug retention of biologic disease modifying antirheumatic drugs in Korean patients with late onset ankylosing spondylitis

**DOI:** 10.1038/s41598-021-01132-6

**Published:** 2021-11-03

**Authors:** Se Hee Kim, Hae-Rim Kim, Sang-Heon Lee, Kichul Shin, Hyoun-Ah Kim, Hong Ki Min

**Affiliations:** 1 Division of Rheumatology, Department of Internal Medicine, Konkuk University Medical Center, 120-1 Neungdong-ro (Hwayang-dong), Gwangjin-gu, Seoul, 05029 Republic of Korea; 2 Division of Rheumatology, Department of Internal Medicine, Konkuk University Medical Center, Konkuk University School of Medicine, Seoul, Republic of Korea; 3 Division of Rheumatology, Department of Internal Medicine, Seoul Metropolitan Government - Seoul National University Boramae Medical Center, Seoul, Korea; 4 Department of Rheumatology, Ajou University School of Medicine, Suwon, Korea

**Keywords:** Rheumatic diseases, Medical research, Rheumatology

## Abstract

The clinical data on the biologic disease-modifying antirheumatic drug (bDMARD) use in late-onset ankylosing spondylitis (LOAS) is limited. Thus, this study aimed to evaluate the drug efficacy and retention rate of bDMARDs in LOAS and compare it to young-onset ankylosing spondylitis (YOAS). Data of patients with AS receiving bDMARDs were extracted from the Korean College of Rheumatology Biologics and Targeted Therapy registry. Patients whose age of onset was > 50 years and ≤ 50 years were classified as having LOAS and YOAS, respectively. Their baseline characteristics and disease-associated parameters were evaluated. Drug efficacy [Ankylosing Spondylitis Disease Activity Score (ASDAS)-clinically important improvement (CII), ASDAS-major improvement (MI), Assessment of SpondyloArthritis International Society (ASAS) 20, and ASAS 40] at 1-year follow-up and drug retention rates were assessed. A total of 1708 patients (comprising 1472 patients with YOAS and 236 patients with LOAS) were included in this analysis. The LOAS group had a lower prevalence among males, lower HLA-B27 positivity and a higher prevalence of peripheral arthritis. Patients with LOAS were more likely to have higher disease-associated parameters (inflammatory reactants, patient global assessment, ASDAS-erythrocyte sedimentation rate, and ASDAS-C-reactive protein). LOAS was negatively associated with achieving ASDAS-CII, ASAS 20, and ASAS 40. The drug retention rate was lower in LOAS; however, the propensity score-matched and covariate-adjusted hazard ratios for bDMARD discontinuation were comparable to YOAS. There were no differences in the drug retention rates based on the type of bDMARD used in LOAS. Inferior clinical efficacy and shorter drug retention time were found in patients with LOAS receiving bDMARDs using real-world nationwide data. There were no differences among each bDMARD type.

## Introduction

Ankylosing spondylitis (AS) is a prototype of axial spondyloarthritis (axSpA), which is a chronic and systemic inflammatory arthritis of the axial skeleton, associated with radiographic changes^1^. Symptoms usually begin before the age of 30 years, and patients are often diagnosed before the age of 40 years. Patients with human leukocyte antigen (HLA)-B27 are more likely to develop axSpA^2^.

Although the Assessment of SpondyloArthritis International Society (ASAS) classification has criteria for inflammatory back pain starting at < 45 years of age^3^, some patients are diagnosed at an older age due to late symptom onset^4^. The issue of patients with late-onset AS (LOAS) raises concerns for revising the definition of inflammatory back pain. Unlike young-onset AS (YOAS), LOAS is less likely to be associated with HLA-B27 positivity, inflammatory back pain, alternating buttock pain, radiographic sacroiliitis, and hip involvement^4–8^. More frequently observed features in LOAS are cervical spine involvement and peripheral arthritis as first manifestations, dactylitis, articular synovitis on power Doppler ultrasound, tenosynovitis or peritendinitis of digit and wrist flexors, and enthesitis of digital collateral ligaments. Additionally, LOAS is associated with female sex, higher levels of acute-phase reactants, nail involvement, and psoriasis^4–6,9^.

AS is usually an early onset disease and is rarely diagnosed in older people. Late-onset disease (symptom onset > 50 years of age) can mimic other rheumatic diseases. This results in a delayed diagnosis of axSpA. Physicians often misdiagnose LOAS as rheumatoid arthritis, polymyalgia rheumatica, and osteoarthritis, as these are more common in the middle age group^10^. The current entry criterion for inflammatory back pain onset < 45 years in axSpA makes diagnosing LOAS challenging and increases the chances of delayed diagnoses and misdiagnoses.

Biologic disease-modifying antirheumatic drugs (bDMARDs) are emerging as promising treatments for AS. Current ASAS/European League Against Rheumatism (EULAR)^11^ and American College of Rheumatology/Spondylitis Association of America/Spondyloarthritis Research and Treatment Network guidelines^12^ recommend their use in patients who do not respond to non-steroidal anti-inflammatory drugs (NSAIDs). The demand for bDMARD prescription would increase if it was shown to have a higher retention rate. However, there is insufficient data on the long-term drug efficacy and retention rate in patients with LOAS^10^.

This study aimed to identify the efficacy and retention rate of bDMARDs in patients with AS > 50 years of age (LOAS) and compare them with the YOAS group in the Korean population. In addition, we also aimed to evaluate the impact of LOAS on drug efficacy and retention rate.

## Methods

### Patient population and data collection

Data were extracted from the Korean College of Rheumatology Biologics and Targeted Therapy (KOBIO) Registry. This is a nationwide prospective cohort that collects data on adverse events and efficacy of biologic and targeted synthetic DMARDs (tsDMARDs) in patients with AS, rheumatoid arthritis, and psoriatic arthritis^13^. A total of 58 tertiary hospital rheumatologic clinics participate in this registry that has been established in 2012. The inclusion criteria for this study were age > 18 years; patients fulfilling the 1984 modified New York (mNY) criteria for AS^14^; and patients who were initiated on, switched to, or re-started on bDMARDs. Patients with non-radiographic axSpA are not enrolled in the KOBIO registry. The patients with AS were divided into LOAS (symptom onset at age > 50 years) and YOAS (symptom onset at age ≤ 50 years) as per previous studies^4,10,15^. Data of patients from the beginning of the registry until October 2020 were included in this study and 2023 patients were initially screened. Exclusion criteria were as follows: (1) patients who lacked follow up data and (2) patients who withdraw informed consent. Informed consent was obtained from all participants before enrolment at each centre. This study was conducted according to the principles of the Declaration of Helsinki and was approved by the Institutional Review Board of Konkuk University Medical Centre (KUMC 2020-12-001).

### Data analysis

Baseline demographics assessed the features of AS and the laboratory information according to the ASAS classification criteria for axSpA , which are inflammatory back pain, arthritis, enthesitis, uveitis, dactylitis, psoriasis, Crohn’s colitis, good response to NSAIDs, family history of SpA, HLA-B27 positivity, and elevated C-reactive protein (CRP). Disease-associated parameters such as Bath Ankylosing Spondylitis Disease Activity Index (BASDAI), Patient’s Global Assessment (PGA), Bath Ankylosing Spondylitis Functional Index (BASFI), Ankylosing Spondylitis Disease Activity Score (ASDAS)-Erythrocyte Sediment Rate (ESR), and ASDAS-CRP were calculated. They were assessed at the 1-year follow-up and changes from baseline were used to assess drug efficacy. This assessment schedule accounted for the fact that longer administration of bDMARDs could precipitate the production of anti-drug antibodies, which could consequently reduce drug efficacy^16,17^. The total bDMARD retention rate was also evaluated. The ASDAS criteria for improvement were ≥ 1.1 units for clinically important improvement (CII) and ≥ 2.0 units for major improvement (MI)^18^. The treatment response was assessed using ASAS 20 and ASAS 40. ASAS 20 was defined as an improvement of least 20% and at least 1 unit in 3 out of 4 domains (PGA, pain assessment, BASFI, and inflammation) on a scale of 0 to 10, without worsening of the remaining domain. ASAS 40 was defined as an improvement of at least 40% and at least 2 units in 3 domains on a scale of 0 to 10, without worsening of the remaining domain^19^. All results of LOAS were compared with those of YOAS.

### Statistical analysis

Continuous variables were initially assessed using the Kolmogorov–Smirnov test to define the normality of distribution. Then Student’s t-test and Mann–Whitney U test were used to compare the continuous variables and present them as mean ± standard deviation (SD) or median [interquartile range (IQR)]. Categorical variables were presented as frequencies and percentages. The χ^2^-test (Mantel–Haenszel χ^2^-test for more than 2 × 2 categorical data) or Fisher’s exact test were used for comparing categorical variables. Logistic regression identified predictors of ASDAS-CII, ASDAS-MI, ASAS 20, and ASAS 40 achievement at 1-year follow-up, and all known covariates potentially influencing drug efficacy were adjusted for. These include sex, body mass index (BMI), HLA-B27 positivity, ASDAS, BASFI, presence of peripheral arthritis, history of bDMARD use (a dichotomous variable; bDMARDs naïve versus bDMARDs exposed), type of bDMARD, and smoking status^20^. The results of the logistic regression analysis were presented as odds ratio (OR) with 95% confidence intervals (CI). Drug retention was analysed using Kaplan–Meier plots and the log-rank test. Cox regression analysis was used to determine the associated variates of bDMARD discontinuation. Propensity score (PS) matching was performed as a 1:1 ratio via imputing variables known to influence drug response. These variables include sex, BMI, smoking status, HLA-B27 positivity, ASDAS, BASFI, peripheral arthritis, and history of bDMARD use. A value of *p* < 0.05 was considered statistically significant. All analyses were performed using SPSS ver. 25 (version 25.0 for Windows, Chicago, IL, USA).

## Results

### Baseline characteristics

A total of 1708 patients with 1-year follow-up data were included in this study, 1472 of whom belonged to the YOAS group and 236 to the LOAS group. The median age was 29.0 years (22.1–37.1 years) and 57.0 years (IQR 54.0–61.1 years) for the YOAS and LOAS groups, respectively. Male predominance was lower in the LOAS group than in the YOAS group (144 patients [61.0%] vs 1170 [79.5%], *p* < 0.001). More patients in the LOAS group had never smoked (140 patients [59.3%] vs 719 patients [48.8%], *p* < 0.001). Patients with LOAS were less likely to be HLA-B27 positive and more likely to have peripheral arthritis. They were also more likely to have higher ESR, CRP, PGA, and ASDAS-ESR/CRP than those with YOAS. In addition, combined co-morbidities (hypertension, ischemic heart disease, hyperlipidemia, osteoporosis, diabetes without complication, hypothyroidism, renal failure, and anemia) were more likely to be present in the LOAS group (Table 1).

**Table 1:** Baseline characteristics of young and late onset ankylosing spondylitis patients at the time of enrollment.

Variable N (%) or median with IQR	YOAS (N = 1472)	LOAS (N = 236)	*p* value^a^
Age (year-old)	29.0 [22.1; 37.1]	57.0 [54.0; 61.1]	< 0.001
Symptom duration (months)	11.6 [30.5; 37.1]	11.4 [32.2; 60.1]	0.092
Male	1170 (79.5%)	144 (61.0%)	< 0.001
BMI	23.3 [21.1; 25.7]	23.6 [21.4; 25.6]	0.456
**Smoking**			< 0.001
Ex-smoker	289 (19.6%)	62 (26.3%)	
Current smoker	464 (31.5%)	34 (14.4%)	
Never	719 (48.8%)	140 (59.3%)	
Biologic naïve	1127 (76.6%)	207 (87.7%)	< 0.001
Right sacroiliitis grade	2.61[2.56; 2.67]	2.59[2.46; 2.72]	0.768
Left sacroiliitis grade	2.6[2.55; 2.65]	2.56[2.43; 2.69]	0.577
Swelling joint count	0.53[0.44; 0.63]	1.59[1.53; 1.65]	0.026
Tender joint count	0.53[0.44; 0.63]	1.11[0.61; 1.61]	0.001
**Features of ASAS Classification Criteria of axSpA**			
Acute Inflammation on MRI highly suggestive of sacroiliitis associated with SpA	332 (22.6%)	61 (25.8%)	0.536
Definite radiographic sacroiliitis according to modified NY criteria	1320 (89.7%)	214 (90.7%)	0.721
Inflammatory back pain	1250 (84.9%)	192 (81.4%)	0.209
Arthritis	392 (26.6%)	95 (40.3%)	< 0.001
Enthesitis	231 (15.7%)	39 (16.5%)	0.847
Uveitis	127 (8.6%)	26 (11.0%)	0.487
Dactylitis	21 (1.4%)	6 (2.5%)	0.414
Psoriasis	38 (2.6%)	8 (3.4%)	0.694
Crohn’s colitis	8 (0.5%)	0 (0.0%)	0.518
Good response to NSAIDs	509 (34.6%)	86 (36.4%)	0.694
Family history for SpA	164 (11.1%)	27 (11.4%)	0.486
HLA-B27	1244 (84.5%)	175 (74.2%)	< 0.001
Elevated CRP	1005 (68.3%)	163 (69.1%)	0.464
**Comorbidity**			
Hypertension	164 (11.1%)	94 (39.8%)	< 0.001
Ischemic Heart Disease	15 (1.0%)	7 (3.0%)	0.002
Hyperlipidemia	185 (12.6%)	47 (19.9%)	0.009
Congestive Heart failure	3 (0.2%)	0 (0.0%)	0.076
Arrhythmia	11 (0.7%)	5 (2.1%)	0.118
Peripheral vascular disorder	0(0%)	0(0%)	1.000
Stroke	2 (0.1%)	0 (0.0%)	1.000
Osteoporosis	38 (2.6%)	29 (12.3%)	< 0.001
Diabetes without complication	36 (2.4%)	30 (12.7%)	< 0.001
Diabetes with complication	7 (0.5%)	2 (0.8%)	0.804
Hyperthyroidism	5 (0.3%)	2 (0.8%)	0.559
Hypothyroidism	7 (0.5%)	9 (3.8%)	< 0.001
Renal failure	6 (0.4%)	6 (2.5%)	0.001
Peptic ulcer	28 (1.9%)	5 (2.1%)	1.000
Liver disease	24 (1.6%)	3 (1.3%)	0.897
Tuberculosis	4 (0.3%)	0 (0.0%)	0.939
Anemia	254 (17.3%)	110 (46.6%)	< 0.001
**Disease associated parameters**			
BASDAI	6.1 [4.8; 7.3]	6.4 [5.0; 7.7]	0.083
PGA	6.3[6.2; 6.4]	6.6[6.3; 6.9]	0.028
ESR	27.5 [13.0; 51.0]	47.5 [20.0; 76.0]	< 0.001
CRP	1.0 [0.3; 2.5]	1.7 [0.4; 4.0]	0.001
ASDAS-ESR	3.6 [2.9; 4.3]	4.1 [3.3; 4.9]	< 0.001
ASDAS-CRP	3.6 [2.9; 4.3]	3.8 [3.0; 4.7]	0.002
BASFI	2.8 [1.0; 5.2]	3.7 [1.8; 6.1]	0.078

IQR, interquartile range; YOAS, Young Onset Ankylosing spondylitis (< 50-year-old); LOAS, Late Onset Ankylosing Spondylitis (≥ 50-year-old); BMI, bone mass index; ASAS, assessment in ankylosing spondylitis; axSpA, axial spondyloarthritis; MRI, magnetic resonance imaging; NY, New York; NSAIDs, non-steroidal anti-inflammatory drugs; CRP, C-reactive protein. BASDAI, Bath ankylosing spondylitis disease activity index; PGA, patient’s global assessment; ASDAS, Ankylosing Spondylitis Disease Activity Score; ESR, erythrocyte sediment rate; BASFI, Bath Ankylosing Spondylitis Functional Index.

^a^Comparing each group with Kruskal–Wallis test or χ^2^ test.

### Drug efficacy at 1-year follow-up

ASDAS-CII, ASDAS-MI, ASAS 20, and ASAS 40 achievement rates at 1-year follow-up for every type of bDMARD are summarised in Table 2. Overall, ASDAS-CII and ASDAS-MI were not different between the two groups, whereas ASAS 20 and ASAS 40 were higher in the YOAS group than in the LOAS group. In the subgroup analysis of specific bDMARDs, there was no significant difference in terms of ASDAS-CII, ASDAS-MI, ASAS 20, and ASAS 40 between the two groups.

**Table 2:** ASDAS-CII and ASDAS-MI in each TNF-α inhibitors.

TNF-α inhibitor		YOAS	LOAS	*p* value^a^
Overall, n (%)	ASDAS-CII	1046 (71.9%)	157 (66.5%)	0.104
	ASDAS-MI	327 (22.5%)	47 (19.9%)	0.424
	ASAS 20	819 (58.9%)	106 (46.7%)	0.001
	ASAS 40	606 (43.6%)	81 (35.7%)	0.031
Etanercept, n (%)	ASDAS-CII	153 (69.9%)	27 (73.0%)	0.851
	ASDAS-MI	54 (24.7%)	10 (27.0%)	0.918
	ASAS 20	113 (51.6%)	15 (40.5%)	0.437
	ASAS 40	78 (35.6%)	13 (35.1%)	0.998
Infliximab, n (%)	ASDAS-CII	221 (68.8%)	41 (62.1%)	0.366
	ASDAS-MI	66 (20.6%)	10 (15.2%)	0.427
	ASAS 20	168 (52.5%)	31 (47.0%)	0.095
	ASAS 40	130 (40.6%)	22 (33.3%)	0.074
Adalimumab, n (%)	ASDAS-CII	423 (72.8%)	64 (68.1%)	0.401
	ASDAS-MI	127 (21.9%)	15 (16.0%)	0.238
	ASAS 20	325 (56.1%)	43 (45.7%)	0.171
	ASAS 40	246 (42.5%)	33 (35.1%)	0.388
Golimumab, n (%)	ASDAS-CII	249 (74.8%)	25 (64.1%)	0.219
	ASDAS-MI	80 (24.0%)	12 (30.8%)	0.474
	ASAS 20	213 (63.4%)	17 (43.6%)	0.055
	ASAS 40	152 (45.2%)	13 (33.3%)	0.356
*p* value^b^	ASDAS-CII	0.317	0.689	
	ASDAS-MI	0.602	0.116	
	ASAS 20	0.076	0.684	
	ASAS 40	0.446	0.688	

^a^Comparing YOAS and LOAS with χ^2^ test.

^b^Comparing between subtypes of TNF-α inhibitors with Mantel–Haenszel χ^2^ test.

YOAS, Young Onset Ankylosing spondylitis (< 50-year-old); LOAS, Late Onset Ankylosing Spondylitis (≥ 50-year-old); ASDAS, Ankylosing Spondylitis Disease Activity Score; CII, Clinically Important Improvement; MI, Major Improvement, ASAS, Assessment of Ankylosing Spondylitis.

The logistic regression analysis showed that LOAS, female sex, and secukinumab were negatively associated with achieving ASDAS-CII. Higher ASDAS-ESR and biologics naïve was positively associated with achieving ASDAS-CII (OR 2.882 and OR 2.004, respectively, Table 3). Higher ASDAS-ESR, higher BASFI, and biologics naïve were positively associated with achieving ASDAS-MI (Supplementary Table S1). LOAS, female sex, BMI < 18.5 kg/m^2^, ex-smoker and current smoker statuses, and HLA-B27 negativity were negatively associated with ASAS 20 response. The same variates (except for smoking status and BMI) were also negatively associated with ASAS 40 response. Higher ASDAS-ESR, higher BASFI, and biologics naïve were positively associated with ASAS 20 and ASAS 40 responses (Supplementary Tables S2 & S3).

**Table 3:** Logistic regression of ASDAS-CII.

Method	Variate	Odds ratio	95% CI	*p* value
Propensity score-based	LOAS (compared to YOAS)	0.507	0.335–0.770	0.001
Covariate adjustment	LOAS (compared to YOAS)	0.497	0.342–0.723	< 0.001
	Female (compared to male)	0.619	0.440–0.870	0.006
	**BMI (kg/m**^**2**^)			
	< 18.5	0.737	0.389–1.393	0.347
	18.5–22.9	1.000 (Reference)		
	23.0–24.9	0.933	0.674–1.291	0.675
	≥ 25.0	0.906	0.671–1.223	0.520
	**Smoking status**			
	Non smoker	1.000 (Reference)		
	Ex-smoker	0.814	0.571–1.161	0.256
	Current smoker	0.765	0.560–1.045	0.093
	HLA B27 negative	0.688	0.465–1.018	0.062
	ASDAS	2.882	2.460–3.377	< 0.001
	BASFI score	1.005	0.950–1.063	0.855
	Peripheral arthritis, yes vs. no	0.812	0.600–1.099	0.177
	Biologics naïve (compared to previous TNFi exposure group)	2.004	1.495–2.681	< 0.001
	**Biologics type**			
	Etanercept	1.000 (Reference)		
	Infliximab	0.837	0.557–1.259	0.393
	Adalimumab	1.175	0.807–1.711	0.401
	Golimumab	1.029	0.675–1.568	0.894
	Secukinumab	0.240	0.064–0.896	0.034

ASDAS, Ankylosing Spondylitis Disease Activity Score; CII, Clinically Important Improvement; YOAS, Young Onset Ankylosing spondylitis (< 50-year-old); LOAS, Late Onset Ankylosing Spondylitis (≥ 50-year-old); BMI, Body Mass Index; ESR, Erythrocyte Sediment Rate; CRP, C-Reactive Protein; BASFI, Bath Ankylosing Spondylitis Functional Index; TNFi, tumour necrosis factor alpha inhibitor.

AS-associated parameters were evaluated using BASDAI, PGA, ESR, CRP, ASDAS-ESR/CRP, and BASFI. The overall scores of disease activity were higher in the LOAS group than in the YOAS group at baseline and 1-year follow-up except for ESR. BASDAI was associated with a lesser decline in the LOAS group than the YOAS group, whereas ESR and CRP were associated with a greater decline in LOAS group. The changes of PGA, ASDAS-ESR/CRP, and BASFI between LOAS and YOAS were not significantly different (Table 4).

**Table 4:** Change of activity at initial enrollment and 1 year follow-up.

	YOAS (N = 1472)	LOAS (n = 236)	*p* value^a^
ΔESR	16.0 [2.5; 38.0]	22.0 [4.0; 54.0]	0.004
ΔCRP	0.7 [0.0; 2.0]	1.2 [0.0; 3.5]	0.005
ΔBASDAI	3.6 [1.7; 5.2]	3.2 [0.9; 4.9]	0.007
ΔPGA	3.0 [1.0; 5.0]	3.0 [1.0; 5.0]	0.059
ΔASDAS-ESR	1.9 [1.0; 2.8]	1.6 [0.8; 2.8]	0.148
ΔASDAS-CRP	2.0 [1.0; 2.9]	1.9 [0.8; 3.0]	0.593
ΔBASFI	1.3 [0.1; 3.5]	1.6 [0.1; 3.8]	0.494

^a^Comparing YOAS and LOAS with Kruskal–Wallis test.

YOAS, Young Onset Ankylosing spondylitis (< 50-year-old); LOAS, Late Onset Ankylosing Spondylitis (≥ 50-year-old); ESR, erythrocyte sediment rate; CRP, C-reactive protein; BASDAI, Bath ankylosing spondylitis disease activity index; PGA, patient’s global assessment; ASDAS, Ankylosing Spondylitis Disease Activity Score; BASFI, Bath Ankylosing Spondylitis Functional Index.

### Drug retention

**Figure 1:**
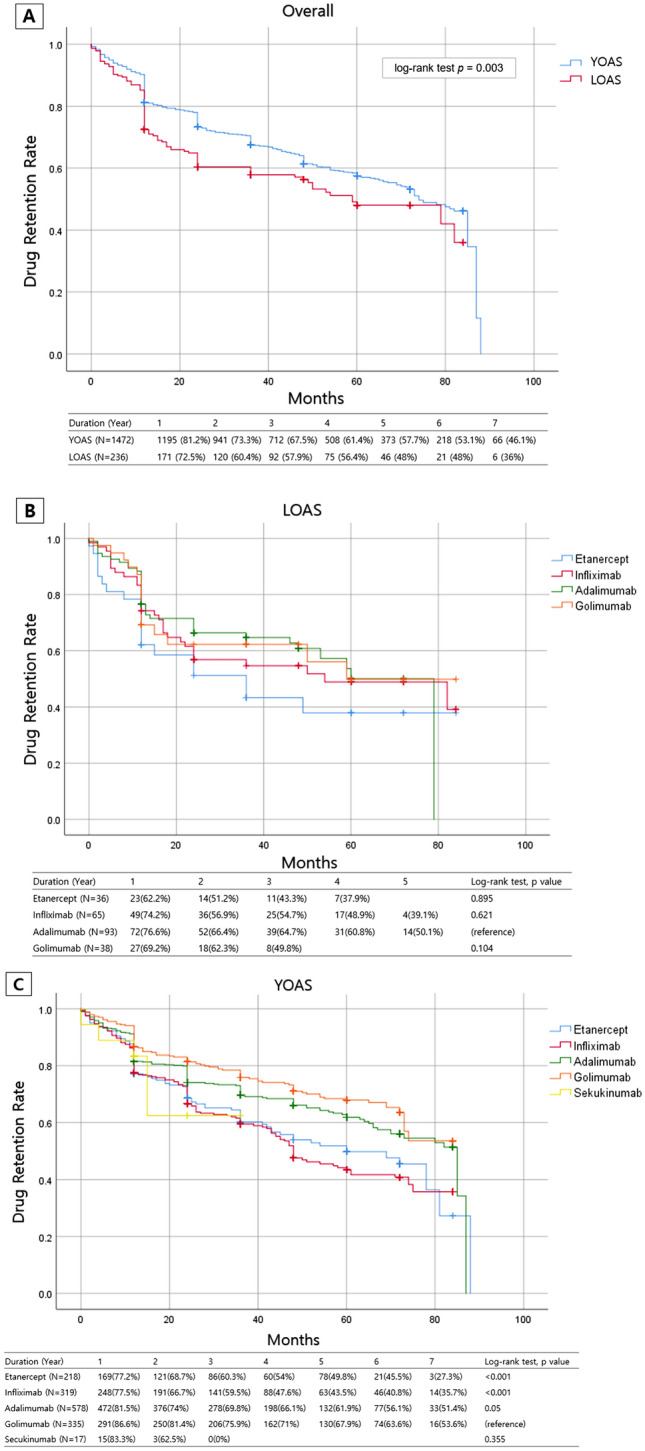
Drug retention rates of biologic disease modifying antirheumatic drug (bDMARD). (**A**) Drug retention rate of overall late onset ankylosing spondylitis (LOAS) and young onset ankylosing spondylitis (YOAS). LOAS showed lower drug retention rate than YOAS. Drug retention rates of each bDMARDs in LOAS (**B**) and YOAS (**C**).

The overall bDMARD retention rates of YOAS and LOAS with median durations of 13.5 months and 11.0 months, respectively are summarised in Fig. 1A. In the LOAS group, the drug retention time was significantly shorter than YOAS (Log-rank test *p* = 0.003). The drug retention rate of LOAS at 1-year follow-up was 72.5%, 57.9% at 3-year, 48.0% at 5-year, and 36% at 7-year follow-up. In YOAS, the drug retention rate at 1-year follow-up was 81.2%, and the rates of every 2 years were 67.5%, 57.7%, and 46.1%, consecutively. Among patients with LOAS (Fig. 1B), adalimumab continuously had the highest and etanercept had the lowest drug retention rates without any significant differences (adalimumab vs golimumab, Log-rank test *p* = 0.895; adalimumab vs infliximab, Log-rank test *p* = 0.621; adalimumab vs etanercept, Log-rank test *p* = 0.104). In the YOAS group (Fig. 1C), golimumab consistently had the highest drug retention rate during the 7 years (golimumab vs etanercept and infliximab, Log-rank test *p* < 0.001; golimumab vs adalimumab, Log-rank test *p* = 0.05). The lowest retention rates were at the 3-year follow-up for secukinumab, 4- to 7-year follow-up for infliximab, without significant differences with other bDMARDs. The most common reasons for discontinuation were inefficacy (LOAS, 38/105 [36.2%]; YOAS, 158/546 [28.9%]) followed by adverse events (LOAS, 31/105 [29.5%]; YOAS, 127/546 [23.3%]) in both groups. In the LOAS group, etanercept demonstrated the highest inefficacy (25.0%) and remission (8.3%) while infliximab had the highest rate of adverse events (16.9%). In the YOAS group, infliximab had the highest inefficiency (15.4%) and etanercept had the highest rate of adverse events (12.4%) (Supplementary Table S4).

### Predictors of bDMARD discontinuation

In the Cox regression analysis of drug discontinuation, LOAS was not significant in the PS-matched or the covariate-adjusted results (HR 1.229; 95% CI 0.931–1.622 and HR 1.184; 95% CI 0.934–1.501, respectively). The HR for bDMARD discontinuation was 1.291 for female sex (95% CI 1.030–1.617), 1.381 for current smokers (95% CI 1.122–1.700), 1.473 for HLA-B27 negativity (95% CI 1.155–1.878), 0.719 for adalimumab (95% CI 0.567–0.9110), and 0.570 for golimumab (95% CI 0.433–0.751) [in reference to etanercept] (Table 5).

**Table 5:** Multivariate Cox regression analysis of bDMARDs discontinuation in patients of ankylosing spondylitis.

Method	Variate	Hazard ratio	95% CI	*p* value
Propensity score-based	LOAS (compared to YOAS)	1.229	0.931–1.622	0.146
Covariate adjustment	LOAS (compared to YOAS)	1.184	0.934–1.501	0.162
	Female (compared to male)	1.291	1.030–1.617	0.027
	**BMI (kg/m**^**2**^)			
	< 18.5	1.263	0.857–1.861	0.239
	18.5–22.9	1.000 (Reference)		
	23.0–24.9	0.997	0.803–1.236	0.975
	≥ 25.0	1.104	0.908–1.344	0.320
	**Smoking status**			
	Non smoker	1.000 (Reference)		
	Ex-smoker	1.116	0.882–1.413	0.361
	Current smoker	1.381	1.122–1.700	0.002
	HLA B27 negative	1.473	1.155–1.878	0.002
	ASDAS	0.945	0.864–1.034	0.216
	BASFI score	1.000	0.965–1.036	0.996
	Peripheral arthritis, yes vs. no	1.178	0.979–1.417	0.082
	Biologics naïve (compared to previous TNFi exposure group)	0.959	0.784–1.172	0.679
	**Biologics type**			
	Etanercept	1.000 (Reference)		
	Infliximab	0.995	0.775–1.277	0.969
	Adalimumab	0.719	0.567–0.911	0.006
	Golimumab	0.570	0.433–0.751	< 0.001
	Secukinumab	0.595	0.185–1.917	0.385

bDMARDs, biologic disease modifying antirheumatic drugs; YOAS, Young Onset Ankylosing spondylitis (< 50-year-old); LOAS, Late Onset Ankylosing Spondylitis (≥ 50-year-old); TNFi, tumour necrosis factor alpha inhibitor; BMI, body mass index; ASDAS, Ankylosing Spondylitis Disease Activity Score; BASFI, Bath Ankylosing Spondylitis Functional Index.

## Discussion

In this study, the efficacy and retention of bDMARDs were examined in patients with LOAS for the first time using a large nationwide registry. Patients with LOAS had a higher prevalence of peripheral arthritis and were less likely to be male or HLA-B27 positive. A lesser decline in BASDAI was observed, in comparison to a greater decline in ESR and CRP in patients with LOAS at the 1-year follow-up compared to those with YOAS. LOAS was negatively associated with achieving ASDAS-CII, ASAS 20, and ASAS 40. The overall drug retention time was shorter in LOAS, without any differences based on bDMARD type. There was no association between LOAS and bDMARD discontinuation.

The use of bDMARDs in older patients with AS is usually associated with poor drug response. In one meta-analysis, older age was negatively associated with achieving BASDAI 50 at the 12-week and 24-week follow-up (OR 0.91, 95% CI 0.84–0.99 and OR 0.98, 95% CI 0.97–0.99, respectively)^21^. The difference between previous studies and ours is that we demonstrated whether LOAS could impact clinical response and bDMARD retention, whereas previous study showed association between age of bDMARDs initiation and efficacy of bDMARDs. Most of the baseline characteristics of LOAS in our study were consistent with those of previous reports^6,15^. In our study, LOAS had higher a prevalence of female sex, peripheral arthritis, ESR, and CRP and a lower prevalence of HLA-B27 positivity compared to YOAS. Moreover, disease-associated parameters of PGA and ASDAS-ESR/CRP were higher in LOAS. Inflammatory back pain was not significantly different between YOAS and LOAS. Several of our findings were different from other reports. Skare et al. found that among those with LOAS, there was a lower prevalence of inflammatory low back pain and uveitis, higher prevalence of dactylitis and psoriasis, and comparable CRP levels compared to that of patients with YOAS^6^. Karaarslan et al. showed that peripheral arthritis was equivalent between LOAS and YOAS^7^. Endo et al. found that LOAS was less likely to be associated with inflammatory back pain and more likely to be associated with dactylitis. It is important to note that they chose the age of 57 years as the differentiating point between YOAS and LOAS^5^. These variations may be due to inclusion criteria differences as our study only enrolled bDMARDs users. Other factors such as ethnic background and symptom duration prior to diagnosis may have also contributed to these differences. Our study enrolled 236 patients with LOAS, which is much higher compared to other studies. Since LOAS has several different characteristics than YOAS, it should be considered as a unique subgroup of patients with AS.

Therapeutic options for LOAS are based on recommendations from the management of younger patients^11,12,15^. Considering the comorbidities are associated with older age, such as peptic ulcer and cardiovascular disease, physicians are often reluctant to prescribe effective drugs such as NSAIDs and TNF-α inhibitors to patients with LOAS. TNF-α inhibitor use in elderly patients with rheumatoid arthritis has shown lower efficacy and increased risk of tuberculosis reactivation, serious infections, and skin cancer^22^. Nonetheless, as far as treating AS, they have shown high efficacy and safety according to the ASAS/EULAR^23^. Moreover, secukinumab has been emerging as a highly effective remedy in these patients^24,25^. Since bDMARDs have been proven efficacious and safe, the demand for this treatment has been increasing. It is important to note that there are mixed results regarding the type of bDMARDs depending on the time of disease onset. Etanercept was found to be well-tolerated and safe in elderly patients with AS^26,27^, whereas the same demographic were more likely to discontinue infliximab and develop severe pyogenic infections^28,29^.

In our study, ASDAS-CII and ASDAS-MI for overall bDMARDs were comparable between LOAS and YOAS; however, ASAS 20 and ASAS 40 were significantly more likely to be achieved in YOAS. In the multivariate logistic regression analysis, we demonstrated that bDMARD use in patients with LOAS patients was negatively associated with achieving clinical response (ASDAS-CII, ASAS20, ASAS40). In addition, objective parameters such as ESR and CRP were associated with a greater decline in LOAS, whereas BASDAI, a subjective patient grading score, was associated with a lesser decline in LOAS. Arends et al. reported that the predictors of achieving clinical response to TNF-α inhibitors were increased acute-phase reactants, higher disease activity, higher functional status, younger age, and HLA-B27 positivity^30^. Rusman et al. reported that female sex was associated with a higher disease activity score, lower quality of life score, and lower response to TNF inhibitors^31^. Several previous studies have found that male sex is a predictor for clinical response and drug retention^32–34^ despite having more severe disease and radiologic damage^31^. It is possible that the clinical response of LOAS could be affected by higher prevalence of female sex or higher level of inflammatory reactant. Therefore we performed PS-matched and multivariate covariate adjusted regression analyses, and being LOAS was still associated with lower clinical response. The lower clinical response and shorter retention time may arise as a consequence of multiple co-morbidities, resulting in polypharmacy, that prompts changes in pharmacokinetics and pharmacodynamics of bDMARDs in the LOAS group^35,36^. However, in vivo measurement of pharmacokinetic and pharmacodynamic interactions between bDMARDs and other medications in patients with LOAS should be unmasked to reveal the mechanisms of lower clinical efficacy and shorter retention time of bDMARDs in the LOAS group.

Similar to previous studies^26–28^, the drug retention time was shorter in the LOAS group than in the YOAS group. However, in the co-variate adjusted and PS-matched results of the Cox regression analysis, LOAS was not significantly associated with drug discontinuation. Although LOAS was negatively associated with achieving clinical efficacy at the 1-year follow- up and had a shorter drug retention time, bDMARDs did diminish disease activity. Therefore, physicians should not hesitate in initiating bDMARDs in LOAS. One of the main strengths of our study is that it was based on real-world data of patients with AS, with relatively large sample size and long follow-up period^5–7^. Moreover, non-radiographic axSpA, which has been shown to be different from AS, was excluded^37^. The results of our study could be specific and helpful for patients with LOAS considering initiating bDMARDs. In addition, the National Health Insurance Service in Korea strictly monitors the diagnosis of AS (through the mNY criteria^14^) and the use of bDMARDs for every rheumatic disease. The initiation, continuation, and discontinuation of bDMARDs are carefully observed as physicians check the disease activity in the designated cycle according to the type of bDMARD. These help in drawing accurate conclusions about the efficacy and drug retention rate of bDMARDs in patients with AS.

Smoking is a well-known aggravating factor for the pathogenesis of AS. Current smokers have more observable progression of spinal structural damage^38^ and lower bDMARDs response^39,40^. However, none of the previous studies demonstrated significant association between smoking status and drug discontinuation of bDMARDs among patients with AS^41,42^. In this study, current smokers had higher risk for bDMARDs discontinuation (HR 1.381, 95% CI 1.233–1.700). This could be meaningful in the sense that smoking could be negatively impacting retention of bDMARDs among patients with AS.

There are some limitations to this study. Firstly, data from KOBIO did not represent the entirety of patients with AS, and only patients receiving bDMARDs were included. Therefore, there could be a difference in baseline characteristics between this group and patients with AS not receiving bDMARDs. Secondly, the pharmacokinetics of each bDMARD was not considered. Each drug has its own time of onset for showing effects in the human body. Disease activity scores and treatment response could not demonstrate the potency of each bDMARD at the first follow-up. Thirdly, the choice of bDMARDs was solely dependent on each rheumatologist’s preference, without a specific protocol. Fourthly, there was no data on secukinumab in LOAS, therefore, its efficacy and retention rate could not be evaluated. Finally, although the cut-off values governing the age range for YOAS and LOAS were reckoned from prior studies^4,10,15^, the cut-off age range for LOAS is still debated. This cut-off age for LOAS (over 50 years old) may consequently influence the results of this study. Despite these limitations, data from the KOBIO registry could reflect the real-world and give useful evidence for starting bDMARDs for patients with LOAS.

In conclusion, patients with LOAS had lower bDMARDs efficacy and drug retention rate when compared to those with YOAS, however being LOAS was not significant factor for predicting drug discontinuation. There were no differences in the efficacy and drug retention rate of each bDMARD in LOAS. These results could be helpful for patients with LOAS who require bDMARD initiation.

## Supplementary Information

Supplementary Tables.
