# Supplementary material for: Effectiveness and drug retention of biologic disease modifying antirheumatic drugs in Korean patients with late onset ankylosing spondylitis
Source: Sci Rep. 2021 Nov 3;11:21555. doi: 10.1038/s41598-021-01132-6 (PMC8566570; doi:10.1038/s41598-021-01132-6)
Supplement: Supplementary file 1 — Supplementary Tables. [file 41598_2021_1132_MOESM1_ESM.docx]

***Supplementary Table***

**Table S1. Logistic regression of ASDAS-MI**

| **Method** | **Variate** | **Odds ratio** | **95% CI** | ***p*-value** |
| --- | --- | --- | --- | --- |
| Propensity score-based | LOAS (compared to YOAS) | 0.781 | 0.504-1.209 | 0.267 |
| Covariate adjustment | LOAS (compared to YOAS) | 0.856 | 0.581-1.262 | 0.433 |
|  | Female (compared to male) | 0.658 | 0.471-0.920 | **0.014** |
|  | BMI (kg/m^2^) |  |  |  |
|  | <18.5 | 0.751 | 0.375-1.503 | 0.419 |
|  | 18.5–22.9 | 1.000 (Reference) |  |  |
|  | 23.0–24.9 | 1.058 | 0.766-1.461 | 0.732 |
|  | ≥25.0 | 1.161 | 0.865-1.558 | 0.321 |
|  | Smoking status |  |  |  |
|  | Non smoker | 1.000  (Reference) |  |  |
|  | Ex-smoker | 0.998 | 0.695-1.433 | 0.991 |
|  | Current smoker | 0.792 | 0.583-1.076 | 0.137 |
|  | HLA B27 negative | 0.691 | 0.468-1.019 | 0.062 |
|  | ASDAS | 1.534 | 1.332-1.767 | **<0.001** |
|  | BASFI score | 1.070 | 1.013-1.130 | **0.015** |
|  | Peripheral arthritis, yes vs. no | 0.396 | 0.134-1.171 | 0.094 |
|  | Biologics naïve (compared to previous TNFi exposure group) | 1.534 | 1.332-1.767 | **<0.001** |
|  | Biologics type |  |  |  |
|  | Etanercept | 1.000  (Reference) |  |  |
|  | Infliximab | 0.645 | 0.429-0.968 | **0.034** |
|  | Adalimumab | 0.711 | 0.495-1.019 | 0.063 |
|  | Golimumab | 0.951 | 0.640-1.413 | 0.803 |
|  | Secukinumab | 0.358 | 0.089-1.443 | 0.149 |

ASDAS, Ankylosing Spondylitis Disease Activity Score; MI, Major Improvement; YOAS, Young Onset Ankylosing spondylitis (<50-year-old); LOAS, Late Onset Ankylosing Spondylitis (≥50-year-old); BMI, Body Mass Index; ESR, Erythrocyte Sediment Rate; CRP, C-Reactive Protein; BASFI, Bath Ankylosing Spondylitis Functional Index; TNFi, tumour necrosis factor alpha inhibitor

**Table S2 Logistic regression ASAS 20**

| **Method** | **Variate** | **Odds ratio** | **95% CI** | ***p*-value** |
| --- | --- | --- | --- | --- |
| Propensity score-based | LOAS (compared to YOAS) | 0.456 | 0.312-0.666 | **<0.001** |
| Covariate adjustment | LOAS (compared to YOAS) | 0.447 | 0.318-0.629 | **<0.001** |
|  | Female (compared to male) | 0.696 | 0.512-0.946 | **0.020** |
|  | BMI (kg/m^2^) |  |  |  |
|  | <18.5 | 0.562 | 0.327-0.967 | **0.037** |
|  | 18.5–22.9 | 1.000 (Reference) |  |  |
|  | 23.0–24.9 | 0.998 | 0.746-1.334 | 0.987 |
|  | ≥25.0 | 0.911 | 0.696-1.193 | 0.497 |
|  | Smoking status |  |  |  |
|  | Non smoker | 1.000  (Reference) |  |  |
|  | Ex-smoker | 0.672 | 0.491-0.920 | **0.013** |
|  | Current smoker | 0.638 | 0.480-0.847 | **0.002** |
|  | HLA B27 negative | 0.689 | 0.475-0.998 | **0.049** |
|  | ASDAS | 1.630 | 1.435-1.851 | **<0.001** |
|  | BASFI score | 1.121 | 1.068-1.178 | **<0.001** |
|  | Peripheral arthritis, yes vs. no | 0.984 | 0.760-1.274 | 0.903 |
|  | Biologics naïve (compared to previous TNFi exposure group) | 1.590 | 1.211-2.083 | **0.001** |
|  | Biologics type |  |  |  |
|  | Etanercept | 1.000  (Reference) |  |  |
|  | Infliximab | 0.977 | 0.678-1.409 | 0.903 |
|  | Adalimumab | 1.075 | 0.771 | 1.499 |
|  | Golimumab | 1.512 | 1.036-2.206 | **0.032** |
|  | Secukinumab | 0.748 | 0.195-2.865 | 0.671 |

ASAS, Assessment of SpondyloArthritis international Society; YOAS, Young Onset Ankylosing spondylitis (<50-year-old); LOAS, Late Onset Ankylosing Spondylitis (≥50-year-old); BMI, Body Mass Index; ASDAS, Ankylosing Spondylitis Disease Activity Score; ESR, Erythrocyte Sediment Rate; CRP, C-Reactive Protein; BASFI, Bath Ankylosing Spondylitis Functional Index; TNFi, tumour necrosis factor alpha inhibitor

**Table S3 Logistic regression ASAS 40**

| **Method** | **Variate** | **Odds ratio** | **95% CI** | ***p*-value** |
| --- | --- | --- | --- | --- |
| Propensity score-based | LOAS (compared to YOAS) | 0.580 | 0.398-0.846 | **0.005** |
| Covariate adjustment | LOAS (compared to YOAS) | 0.557 | 0.395-0.785 | **0.001** |
|  | Female (compared to male) | 0.729 | 0.538-0.989 | **0.042** |
|  | BMI (kg/m^2^) |  |  |  |
|  | <18.5 | 0.733 | 0.425-1.265 | 0.265 |
|  | 18.5–22.9 | 1.000 (Reference) |  |  |
|  | 23.0–24.9 | 0.934 | 0.702-1.244 | 0.642 |
|  | ≥25.0 | 0.849 | 0.650-1.109 | 0.230 |
|  | Smoking status |  |  |  |
|  | Non smoker | 1.000  (Reference) |  |  |
|  | Ex-smoker | 0.905 | 0.664-1.233 | 0.526 |
|  | Current smoker | 0.783 | 0.592-1.036 | 0.087 |
|  | HLA B27 negative | 0.664 | 0.449-0.981 | **0.040** |
|  | ASDAS | 1.603 | 1.413-1.817 | **<0.001** |
|  | BASFI score | 1.117 | 1.065-1.171 | **<0.001** |
|  | Peripheral arthritis, yes vs. no | 0.964 | 0.749-1.242 | 0.778 |
|  | Biologics naïve (compared to previous TNFi exposure group) | 1.412 | 1.072-1.862 | **0.014** |
|  | Biologics type |  |  |  |
|  | Etanercept | 1.000  (Reference) |  |  |
|  | Infliximab | 1.130 | 0.781-1.635 | 0.518 |
|  | Adalimumab | 1.206 | 0.863-1.686 | 0.273 |
|  | Golimumab | 1.312 | 0.904-1.905 | 0.153 |
|  | Secukinumab | 0.213 | 0.025-1.845 | 0.160 |

ASAS, Assessment of SpondyloArthritis international Society; YOAS, Young Onset Ankylosing spondylitis (<50-year-old); LOAS, Late Onset Ankylosing Spondylitis (≥50-year-old); BMI, Body Mass Index; ASDAS, Ankylosing Spondylitis Disease Activity Score; ESR, Erythrocyte Sediment Rate; CRP, C-Reactive Protein; BASFI, Bath Ankylosing Spondylitis Functional Index; TNFi, tumour necrosis factor alpha inhibitor

Table S4 Reasons for discontinuation of bDMARDs in YOAS and LOAS

|  | **Etanercept** | | **Infliximab** | | **Adalimumab** | | **Golimumab** | | **Secukinumab** | |
| --- | --- | --- | --- | --- | --- | --- | --- | --- | --- | --- |
|  | YOAS  (N=218) | LOAS  (N=36) | YOAS  (N=319) | LOAS  (N=65) | YOAS  (N=578) | LOAS  (N=93) | YOAS  (N=335) | LOAS  (N=38) | YOAS  (N=17) | LOAS  (N=0) |
| Remission, n(%) | 6 (2.8%) | 3 (8.3%) | 14 (4.4%) | 2 (3.1%) | 28 (4.8%) | 5 (5.4%) | 10 (3.0%) | 2 (5.3%) |  |  |
| Inefficacy, n(%) | 21 (9.6%) | 9 (25%) | 49 (15.4%) | 13 (20.0%) | 47 (8.1%) | 11 (11.8%) | 40 (11.9%) | 5 (13.2%) | 1 (5.9%) |  |
| Adverse event, n(%) | 27 (12.4%) | 4 (11.1%) | 35 (11.0%) | 11 (16.9%) | 47 (8.1%) | 11 (11.8%) | 17 (5.1%) | 5 (13.2%) | 1 (5.9%) |  |
| Other reason, n(%) | 39 (17.9%) | 4 (11.1%) | 54 (16.9%) | 6 (9.2%) | 74 (12.8%) | 10 (10.8%) | 34 (10.1%) | 4 (10.5%) | 2 (11.8%) |  |

bDMARDs, biologic disease modifying antirheumatic drugs; YOAS, Young Onset Ankylosing spondylitis (<50-year-old); LOAS, Late Onset Ankylosing Spondylitis (≥50-year-old)
